# Pitfalls in the Diagnosis of Primary Hyperparathyroidism in a Sudanese Adolescent Boy; a case disguised as rickets

**DOI:** 10.1186/s12902-022-01241-x

**Published:** 2022-12-19

**Authors:** Sara MohammedAhmed Gafar, Ghassan Faisal Fadlalbari, Asmahan T. Abdalla, Sawsan Abdel Rahim Mohammed, Mohammed Khalid Alrasheed, Isam Ahmed Taha, Mohamed Ahmed Abdullah

**Affiliations:** 1 Department of Pediatric Endocrinology, Gaafar Ibn Auf Pediatric Tertiary Hospital, Khartoum, Sudan; 2 Sudan Childhood Diabetes Center, Khartoum, Sudan; 3 Department of Pathology, Faculty of Medicine, University of Khartoum, Khartoum, Sudan; 4 Department of Orthopaedics and Traumatology, Faculty of Medicine, University of Khartoum, Khartoum, Sudan; 5 Department of pediatric surgery, National Ribat Hospital & Faculty of Medicine, National Ribat University, Khartoum, Sudan; 6 Department of Pediatrics and Child Health, Faculty of Medicine, |University of Khartoum, Khartoum, Sudan

**Keywords:** Primary hyperparathyroidism, Parathyroid adenoma, Genu valgum, Adolescents, Vitamin D deficiency

## Abstract

**Background:**

Juvenile primary hyperparathyroidism (PHPT) is a rare endocrine disease. Its diagnosis might be masked by clinical, biochemical, and radiological features of rickets.

**Case presentation:**

A 12-year-old Sudanese boy presented with progressive lower limbs deformity and difficulty in walking for six months. It was associated with fatigability, poor appetite, and generalized bone pain. On examination, he was thin, disproportionately short and pubertal, and had bilateral genu valgum deformity. X-rays showed osteopenia and signs of rickets. Biochemical workup revealed mildly elevated serum calcium, low phosphate, high alkaline phosphatase, and high parathyroid hormone with low 25-hydroxy vitamin D_3_. Celiac screening, liver function test and renal profile were normal. Serum calcium rose dramatically after vitamin D therapy. Genetic testing was negative for CYP2R1 and MEN1 genes. Ultrasound neck showed left inferior parathyroid adenoma which was surgically excised. Histopathology confirmed the diagnosis of parathyroid adenoma. Postoperatively, he had hypocalcemia which was treated with calcium and alfacalcidol. Corrective surgery is planned for the genu valgum deformity which markedly improved after parathyroidectomy.

**Conclusion:**

Although PHPT is extremely rare in the young population, it should be considered in patients with rickets and elevated serum calcium at baseline or after initiating vitamin D therapy.

## Background

Primary hyperparathyroidism (PHPT) is a rare endocrine disorder in children and adolescents [1]. Its incidence is estimated to be 1/200–300,000 compared to 28/100,000 in adult patients [2, 3]. Until 2012, PHPT was reported in around 268 children and adolescents in the literature [4]. Unlike in adults in whom presentation is usually asymptomatic and only diagnosed by accidental detection of hypercalcemia, children and adolescents with PHPT usually present with end-organ damage. That is due to the non-specific presenting symptoms which lead to delay in diagnosis and management [5]. We herein report an adolescent boy with PHPT who presented with skeletal and radiological manifestations of rickets.

## Case presentation

**Fig. 1:**
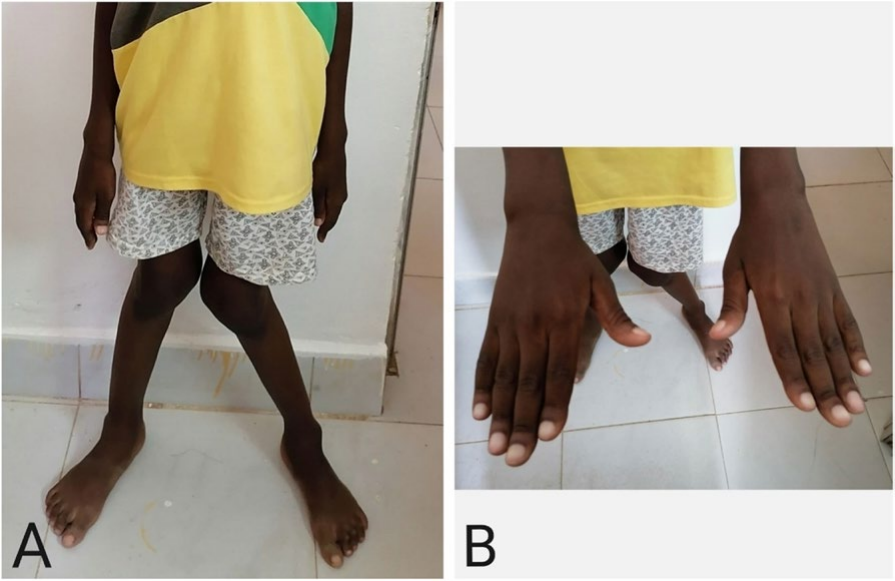
**A:** Genu valgus and wrist widening, **B:** finger pseudo-clubbing

**Fig. 2:**
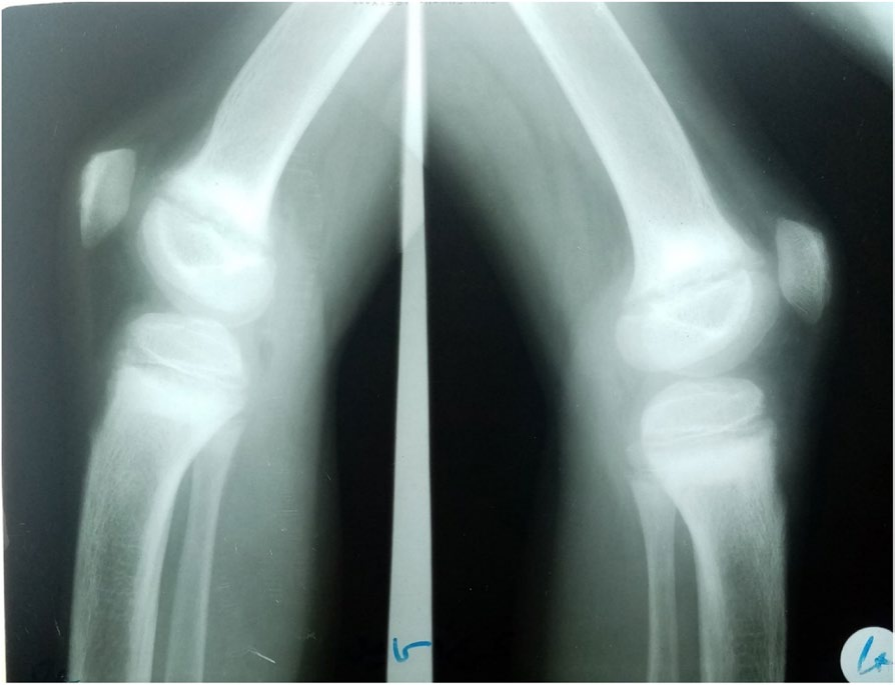
X-ray bilateral knees shows osteopenia, mild cupping, and fraying

A 12-year-old Sudanese boy presented with progressive lower limb deformity and difficulty in walking for six months. The condition was associated with generalized fatigability, poor appetite, bone pain, headache, and change in behavior and deterioration in school performance. However, he did not complain of abdominal pain, constipation, polyuria or polydipsia. He was previously healthy and participating in school sports activities until six months prior to presentation to our facility. There were no symptoms suggestive of malabsorption or renal disease and had neither history of trauma nor fractures. He had an average birth weight, went through a normal neonatal period and developmental milestones. Sun exposure was adequate and the diet was balanced. He is an outcome of non-consanguineous marriage and has two healthy sisters. His mother had a renal stone which was treated conservatively, otherwise, there is no family history suggestive of multiple endocrine neoplasias (MEN) or vitamin D pathway defect. He received therapeutic doses of vitamin D_3_ for three months after being diagnosed to have vitamin D deficiency rickets by his treating physician at a primary health care facility (Fig. 1 & 2, Table 1). Thereafter, he was referred to our Endocrinology clinic for further management when he showed no response to medical therapy.

**Table 1:** Biochemical workup of the patient on presentation, pre-and postoperatively

Parameter (normal references)	Baseline	At our unit	1 day Pre OP^*^	5 days Post OP^+^	21 days Post OP^+^	Last visit
	6/12/2019	25/2/2020	15/10/2020	20/10/2020	5/11/2020	7/6/2021
Serum Ca^+2^ (8.1–10.4 mg/dl)	11.7	14	10.7	7.8	6.6	8.7
Serum Po^+2^ (3.5–5.5 mg/dl)		2.7				5.1
ALP (48–162 IU/L)		5556				352
25-hydroxyvitamin D (> 30 ng/dl)	8.1	12				
Creatinine		0.3				
GFR (mL/min/1.73m^2^)		120				
PTH^†^ (15–65 pg/ml)		3509				45
Urine Ca/Cr^††^ ratio (< 0.20 mg:mg)		0.65				

^*^Preoperative,^+^ Postoperative, ^†^ Parathyroid Hormone, ^††^ Calcium to Creatinine

On presentation to us, he was thin (−2.0 SD), disproportionately short (−3.0 SD), pubertal (Tanner II genitalia) and there was no dysmorphism. He had bilateral genu valgum deformity that was more marked on the left side, positional scoliosis, non-tender rachitic rosaries, pseudo-clubbing and wide wrists with double malleoli (Fig. 1). The rest of the systemic examination was unremarkable. Laboratory tests revealed a normal Complete Blood Count (CBC) as well as normal Liver Function Tests (LFT), and negative celiac screening. Calcium oxalate was detected on urinalysis in addition to hypercalcemia, low phosphate, markedly elevated alkaline phosphatase and high parathyroid hormone (PTH) with a low 25 (OH) vitamin D_3_ level (Table 1). He was admitted to our hospital to investigate and manage hypercalcemia. Basic electrocardiography (ECG) showed no arrhythmias. He received intravenous saline and loop diuretics. Two days later, he was started on cinacalcet 30 mg PO q 12 hours after which serum calcium dropped from 14.0 to 11.0 mg/dl.

**Fig. 3:**
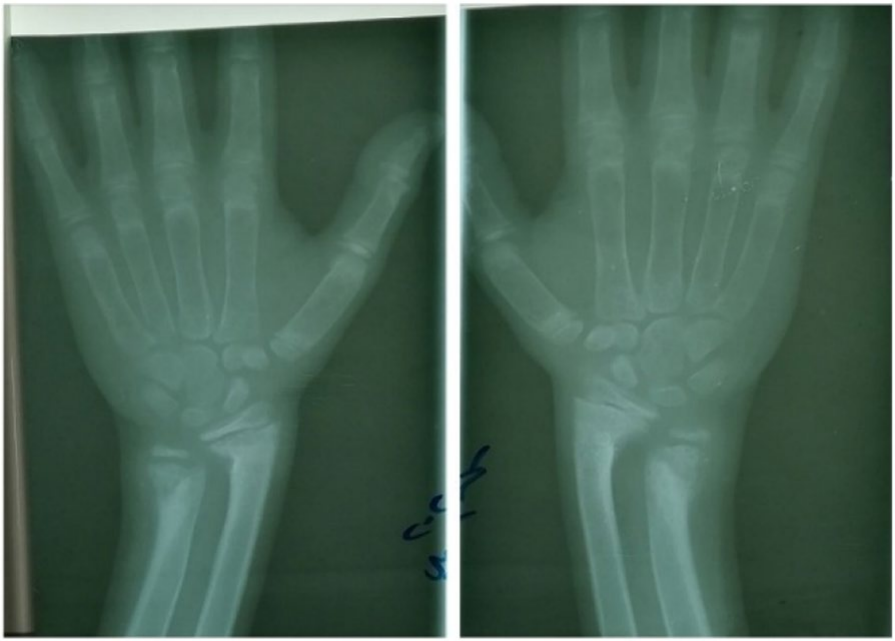
X-ray bilateral wrist illustrates angulation deformity of distal ends of both ulna and radius

He was also investigated for renal involvement which showed normal renal function, high urinary calcium: creatinine ratio with no evidence of nephrocalcinosis or renal stones on the renal ultrasonography (Table 1). Bilateral knees x-ray showed osteopenia, mild cupping, and fraying while his upper limbs x-ray showed severe osteopenia with angulation deformity at the distal end of both radius and ulna bilaterally (Fig. 2 & 3). Later on, the duodenal biopsy specimen did not show any feature of celiac disease. Neck ultrasound demonstrated 23x8 mm left inferior parathyroid adenoma and normal thyroid gland. Tc 99 Sestamibi scan was not done for financial constraints. Whole-exome sequencing was done to rule out CYP2R1 and MEN1 which were reported negative.

**Fig. 4:**
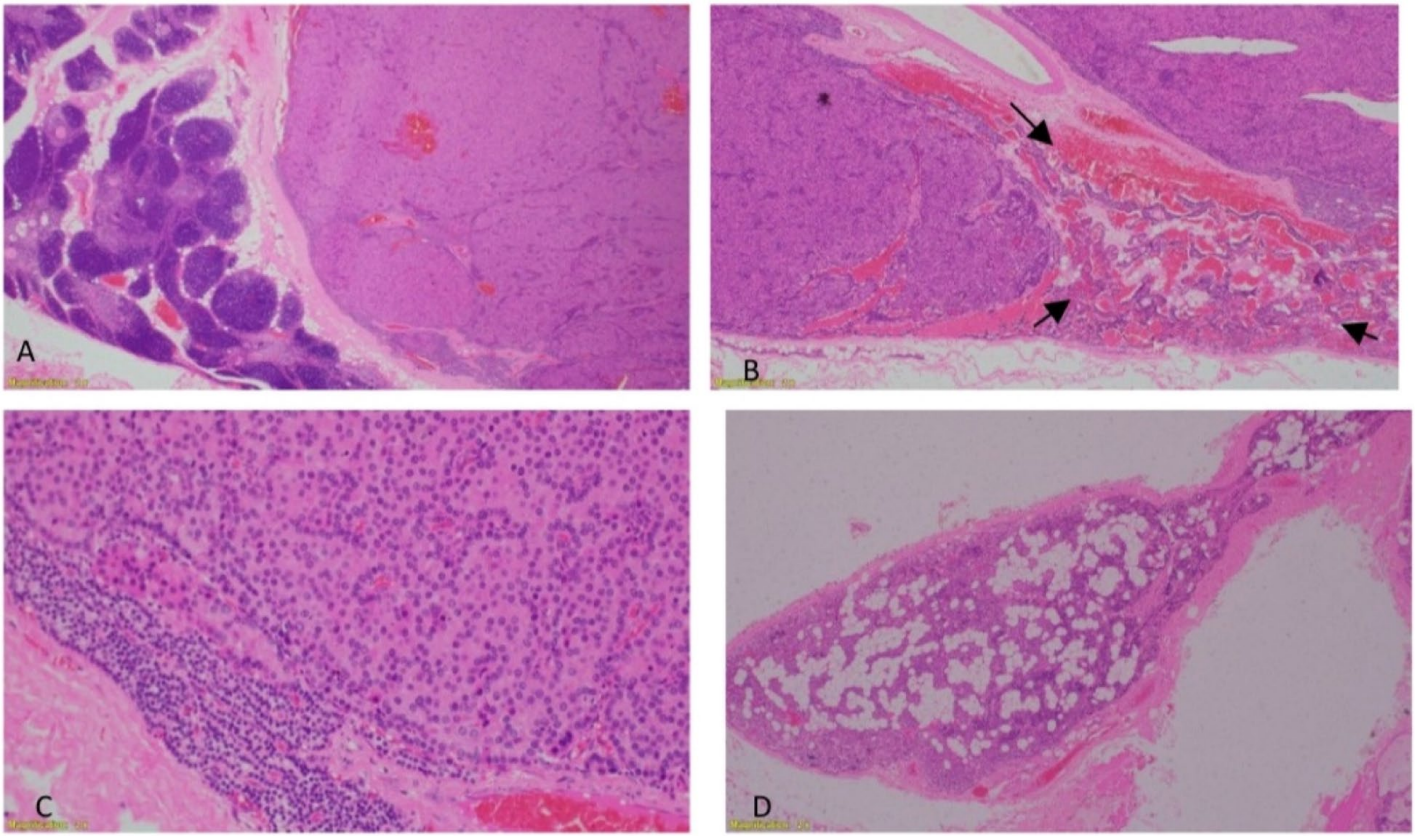
**A:** Left inferior parathyroid adenoma with attached normal thymic tissue on the left x20, **B:** Left inferior parathyroid adenoma shows compressed atrophic rim of parathyroid tissue (lower right arrows) x40, **C:** parathyroid adenoma with compressed atrophic rim of parathyroid tissue x200, **D:** Left upper parathyroid shows adipose tissue indicative of atrophic changes x20

**Fig. 5:**
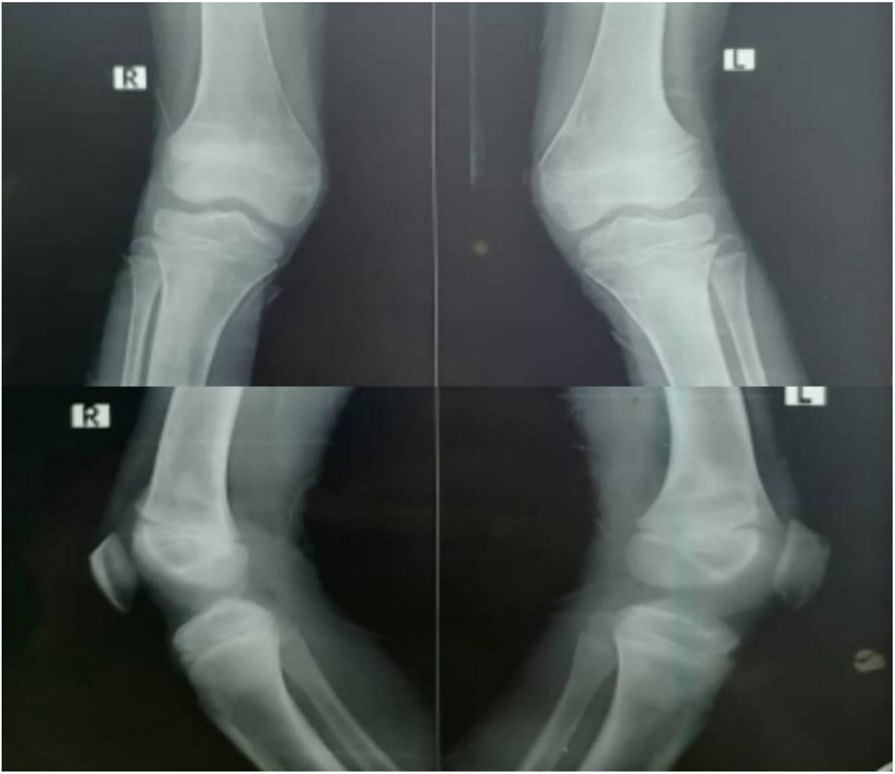
X-ray bilateral knees shows improvement of osteopenia and healing of rickets signs after surgery

The patient was planned for surgery and started on alfacalcidol two days preoperatively. The left inferior parathyroid gland was removed. Histopathology showed parathyroid adenoma composed of compact nests of polygonal chief cells with clear cytoplasm. There was a rim of compressed atrophic parathyroid tissue (Fig. 4). Postoperatively, the patient developed hypocalcemia which was corrected by oral calcium and alfacalcidol. Subsequent follow-up showed normalization of serum calcium, phosphate and a significant drop in alkaline phosphatase. He was weaned successfully from calcium and alfacalcidol (Table 1). He was seen eight months after parathyroidectomy during follow up, he was more energetic and able to walk longer distances following the improvement on his lower limbs deformity and muscular built (Fig. 5). His residual left side genu valgum is planned to be surgically corrected using hemiepiphysiodesis.

## Discussion and Conclusions

Diagnosis of PHPT in the young population might be a challenge because it is uncommon and usually presents with vague symptoms in this age group [2, 5]. In contrast to neonates, in whom PHPT presents commonly with neurological symptoms and can be detected earlier, older children with PHPT usually have end-organ damage and skeletal manifestations at presentation [6].

The initial diagnosis of rickets was suggested by the clinical and radiological features of active rickets and supported by the laboratory findings of markedly elevated alkaline phosphatase, high PTH and low vitamin D level. However, development of marked hypercalcemia with persistently high PTH level upon receiving therapeutic doses of vitamin D3 prompted us to consider PHPT rather than rickets with secondary hyperparathyroidism. His very high level of serum calcium and parathyroid hormone lead us to consider parathyroid carcinoma but the size of the gland, absent of thyroid involvement and the histopathology confirmed the diagnosis of parathyroid adenoma. This led us to the query that whether clinical and radiological rachitic signs are part of the PHPT skeletal manifestations or were due to vitamin D deficiency complicating PHPT. It has been argued that PHPT among the young population is more common in vitamin D deficient areas [7, 8]. It has also been shown that elevated PTH increases vitamin D hepatic clearance shortening its half-life duration [8]. Furthermore, some authors suggested that a high level of PTH increases the conversion of 25 (OH) vitamin D to 1,25 (OH) vitamin D contributing to the low level of 25 (OH) vitamin D, though it does not rationalize the presence of the rachitic features with a sufficiency of vitamin D active form [8]. Therefore, low vitamin D in our patient might be explained by his excess PTH. Yet, poor sun exposure due to walking limitation and being indoor most of the time secondary to his lower limbs deformity is a definite risk factor.

Although genu valgum is a well-known skeletal manifestation in patients with rickets, in 2014 Ramkumar et al. reported 13 cases of PHPT who had genu valgum as a presenting feature [9]. Since then, additional cases have appeared in the literature indicating an etiological link between PHPT and genu valgum [5, 7, 10]. All reported cases of genu valgum associated with PHPT, including our patient, are adolescents with rapid growth spurt which may reflect a direct effect of PTH on the growth plate during this period. Furthermore, none of them had a brown tumor at the growth plate to explain the development of genu valgus [5, 7, 10]. Beside genu valgum deformity, our patient also had angulation deformities at the ends of the radius and ulna that can be explained by excess use of upper limbs as an aid to get up from a sitting position to overcome the lower limbs weakness and pain.

It is worth mentioning here that the coexistence of vitamin D deficiency exaggerates bone disease in PHPT patients [11–13]. It has been associated with delayed bone recovery, hunger bone syndrome and secondary hyperparathyroidism postoperatively [8, 11, 13]. Huai Heng et al. found that vitamin D supplementation in patients with mild PHPT improves 25 (OH) vitamin D levels without worsening of hypercalcemia or hypercalciuria [14]. To the best of our knowledge, there are no studies done to detect the effect of vitamin D treatment in patients with severe PHPT.

In conclusion, rickets can be a feature of PHPT in an adolescent. One should consider PHPT in any child/adolescent who lack clinical and radiological response to conventional therapy of vitamin D3, had hypercalcemia at initial presentation or following vitamin D3 supplement, Early diagnosis of PHPT is important for proper pre and post-operative management.

## Data Availability

All data are included in this manuscript.

## Abbreviations

CYP2R1: Cytochrome PR1 gene
MEN: Multiple endocrine neoplasias
PHPT: Primary hyperparathyroidism
PO: Per oral
PTH: Parathyroid hormone
Tc99: Technetium 99
